# Supplementary material for: Inhibition of Advanced Glycation End-Products by Tamarindus indica and Mitragyna inermis Extracts and Effects on Human Hepatocyte and Fibroblast Viability
Source: Molecules. 2023 Jan 2;28(1):393. doi: 10.3390/molecules28010393 (PMC9823519; doi:10.3390/molecules28010393)
Supplement: Supplementary file 1 [file molecules-28-00393-s001.zip › molecules-2076820-supplementary.pdf]

molecules

MDPI

Supplementary Materials

Article

Inhibition of Advanced Glycation End-Products by *Tamarindus indica* and *Mitragyna inermis* Extracts and Effects on Human Hepatocyte and Fibroblast Viability

Relwendé Justin Ouédraogo [1,2,*], Umair Aleem [3], Lassina Ouattara [1,2], Muhammad Nadeem-ul-Haque [4], Georges Anicet Ouédraogo [1,2], Humera Jahan [3,*] and Farzana Shaheen [4,*]

[1] Department of Biochemistry-Microbiology, Unit of Training and Research in Life and Earth Sciences, Nazi Boni University, Bobo-Dioulasso 01 BP 1091, Burkina Faso
[2] Laboratory of Research and Teaching in Animal Health and Biotechnology, Nazi Boni University, Bobo-Dioulasso 01 BP 1091, Burkina Faso
[3] Dr. Panjwani Center for Molecular Medicine and Drug Research, International Center for Chemical and Biological Sciences, University of Karachi, Karachi 75270, Pakistan
[4] Third World Center for Science and Technology, Hussain Ebrahim Jamal Research Institute of Chemistry, University of Karachi, Karachi-75270, Pakistan
* Correspondence: rjustino14@yahoo.com (R.J.O.), jahan_pcmd@yahoo.com (H.J.); afnan.iccs@gmail.com (F.S.); Tel.: +226-75386260 (R.J.O.); +92-21-111-232-292 (H.J.); +92-331-3859073 (F.S.)

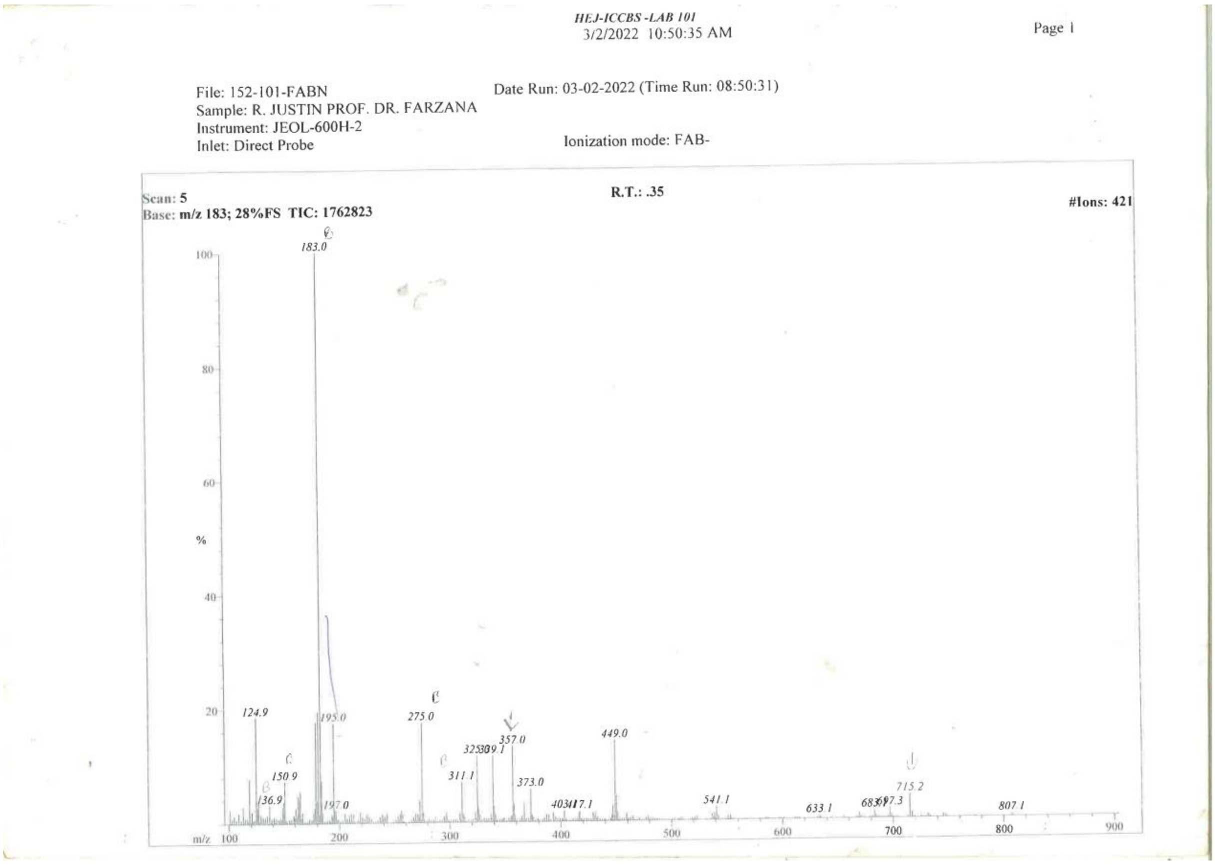

Figure S1: FABMS spectrum of compound **1**

HEJ-MASS LAB -ICCBS		JEOL HX 110 MASS SPECTROMETER (FAB-HR)		
STUDENT NAME	R. JUSTIN	SAMPLE CODE	DATE	7/3/2022
SUPERVISOR NAME	DR. FARZANA SHAHEEN	R-152-101	FAB (+VE / -VE)	+VE MODE

Measured Mass	Theoretical Mass	Delta [ppm]	Delta [mmu]	RDB	Composition
359.1511	359.1495	4.6	1.6	9.5	C20 H23 O6
	359.1553	-11.8	-4.2	0.5	C13 H27 O11
	359.1436	20.9	7.5	18.5	C27 H19 O1
357.1355	357.1338	4.7	1.7	10.5	C20 H21 O6
	357.1397	-11.7	-4.2	1.5	C13 H25 O11
	357.1279	21.2	7.6	19.5	C27 H17 O1

Figure S2: HRFABMS spectrum of compound **1**

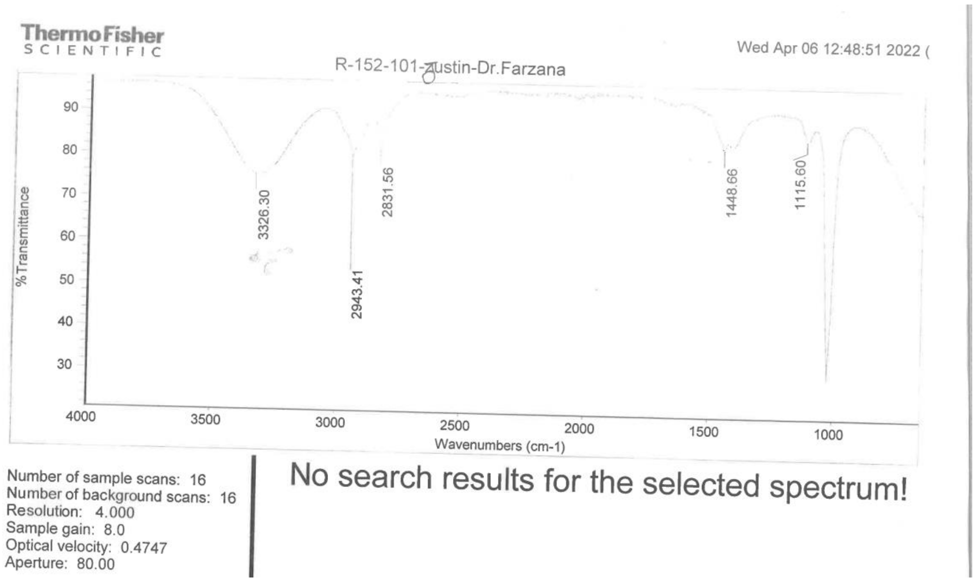

Figure S3 : IR spectrum of compound **1**

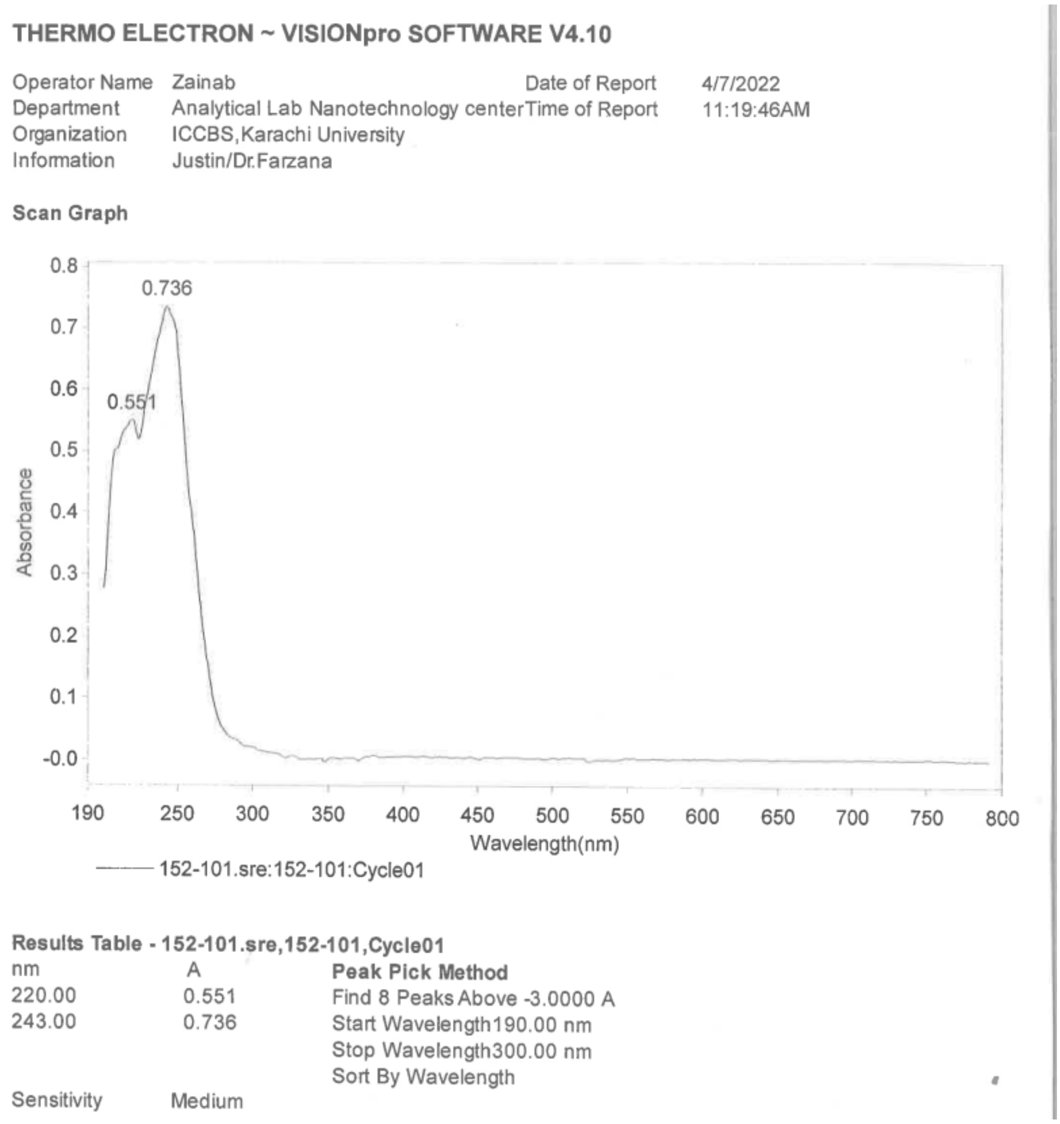

Figure S4 : UV/UV-Visible (MeOH) spectrum of compound **1**

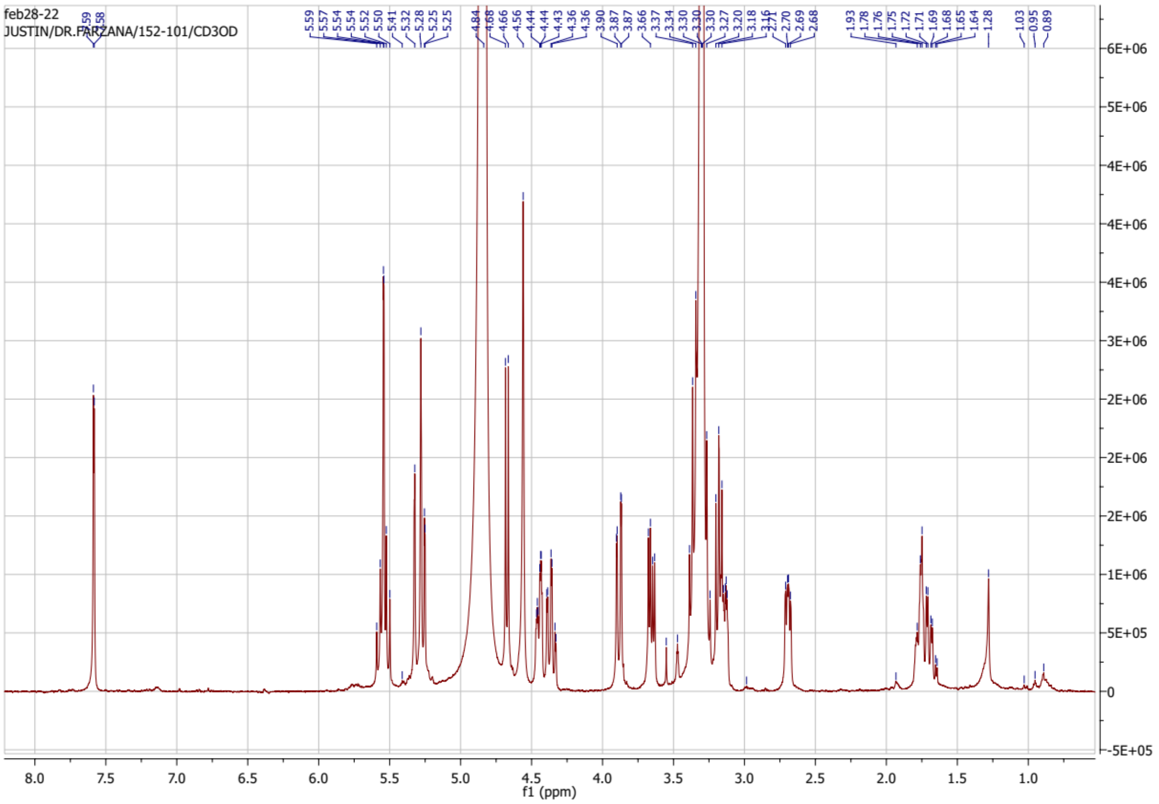

Figure S5: ^{1}H NMR (400 MHz, CD_3OD) spectrum of compound **1**

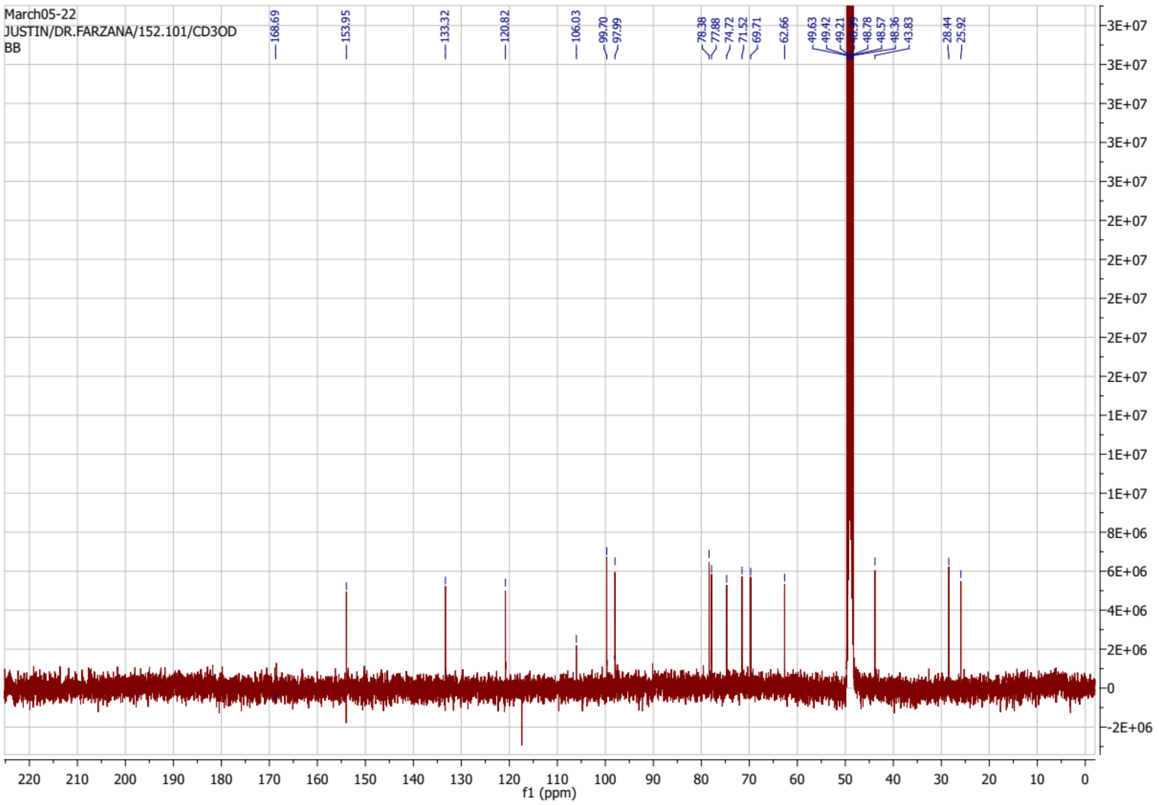

Figure S6: ^{13}C NMR (101 MHz, CD_3OD) spectrum of compound **1**

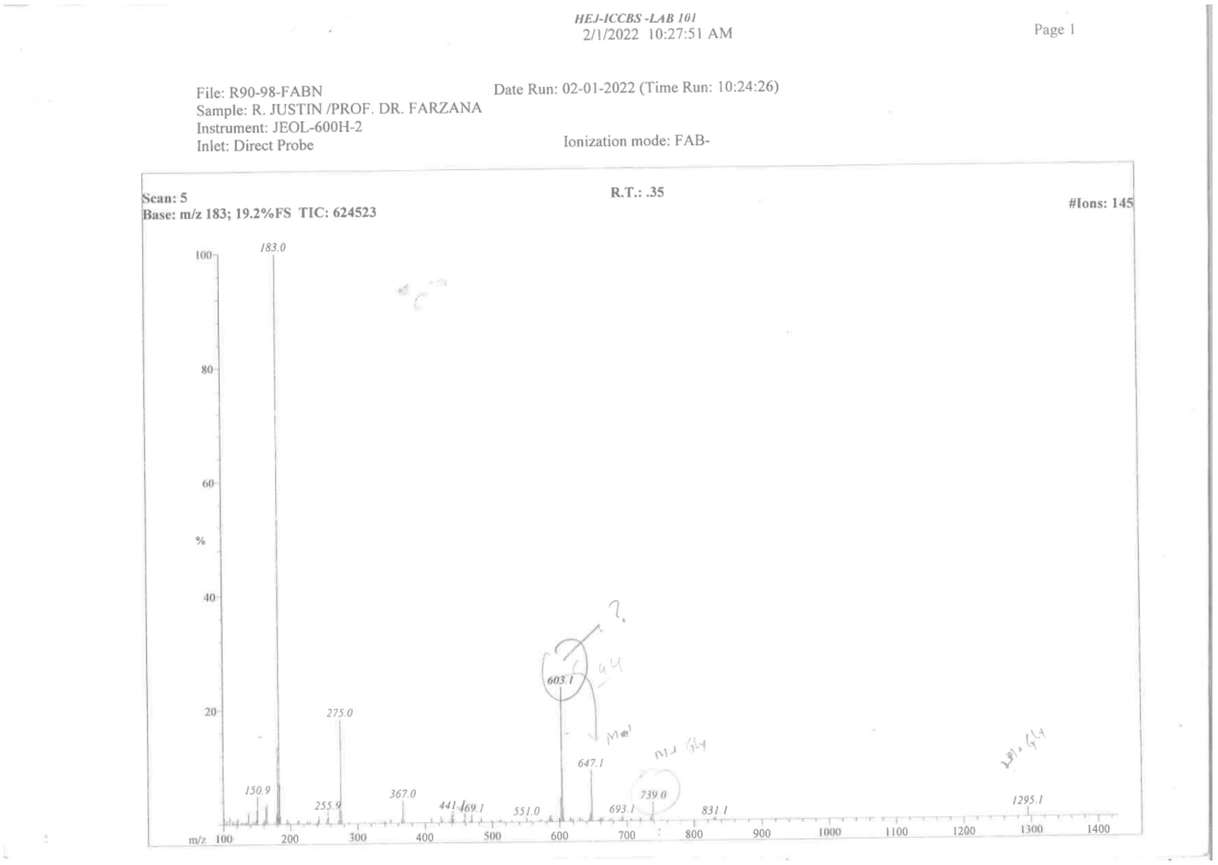

Figure S7: FABMS spectrum of compound **2**

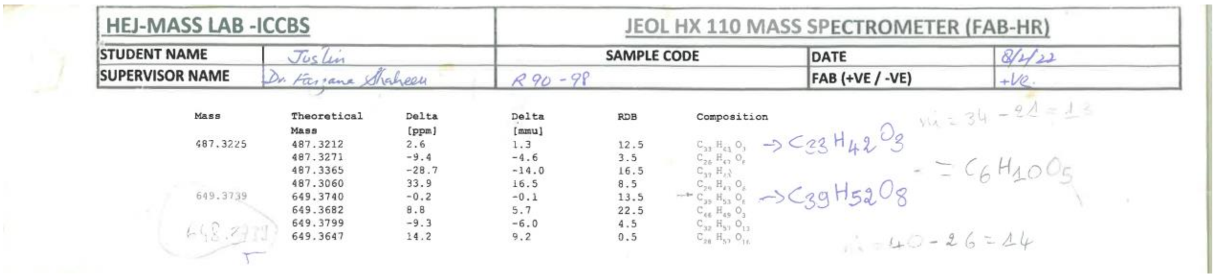

HEJ-MASS LAB -ICCBS		JEOL HX 110 MASS SPECTROMETER (FAB-HR)		
STUDENT NAME	Justin	SAMPLE CODE	DATE	8/4/22
SUPERVISOR NAME	Dr. Farzana Shaheen	R 90 - 98	FAB (+VE / -VE)	+ve

Mass	Theoretical Mass	Delta [ppm]	Delta [mmu]	RDB	Composition
487.3225	487.3212	2.6	1.3	12.5	$C_{33}H_{43}O_3$
	487.3271	-9.4	-4.6	3.5	$C_{26}H_{47}O_8$
	487.3365	-28.7	-14.0	16.5	$C_{37}H_{43}$
	487.3060	33.9	16.5	8.5	$C_{29}H_{43}O_6$
649.3739	649.3740	-0.2	-0.1	13.5	$C_{39}H_{53}O_8$
	649.3682	8.8	5.7	22.5	$C_{46}H_{49}O_3$
	649.3799	-9.3	-6.0	4.5	$C_{32}H_{57}O_{13}$
	649.3647	14.2	9.2	0.5	$C_{28}H_{57}O_{16}$

Figure S8: HRFABMS spectrum of compound **2**

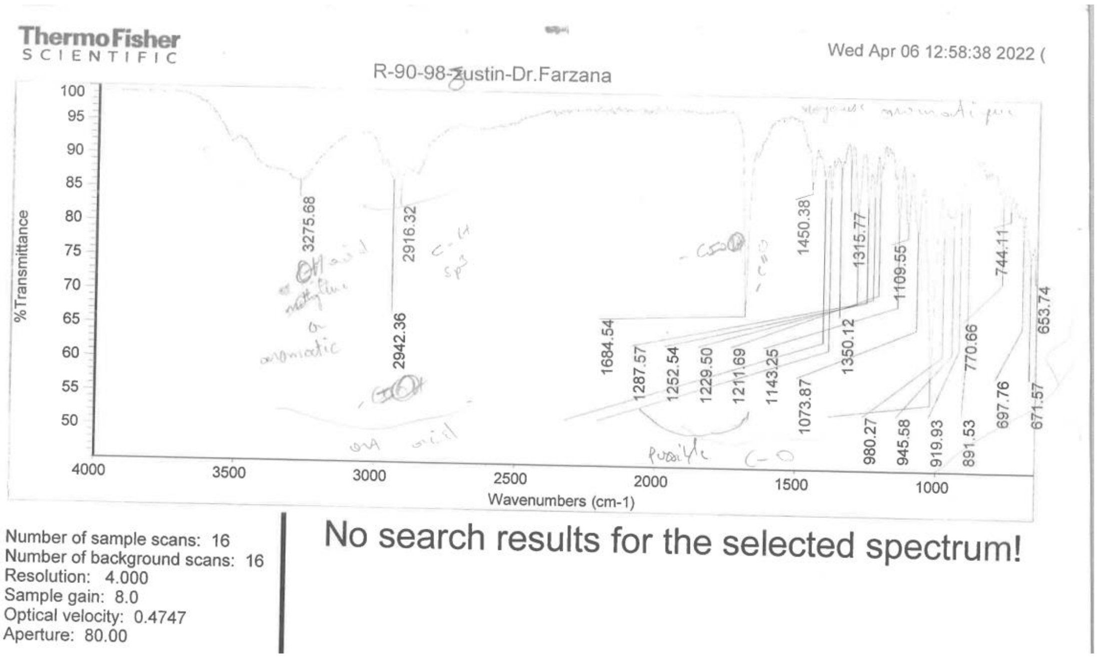

Figure S9: IR spectrum of compound **2**

THERMO ELECTRON ~ VISIONpro SOFTWARE V4.10

Operator Name	Zainab	Date of Report	4/7/2022
Department	Analytical Lab Nanotechnology center	Time of Report	11:17:43AM
Organization	ICCBS,Karachi University		
Information	Justin/Dr.Farzana		

Scan Graph

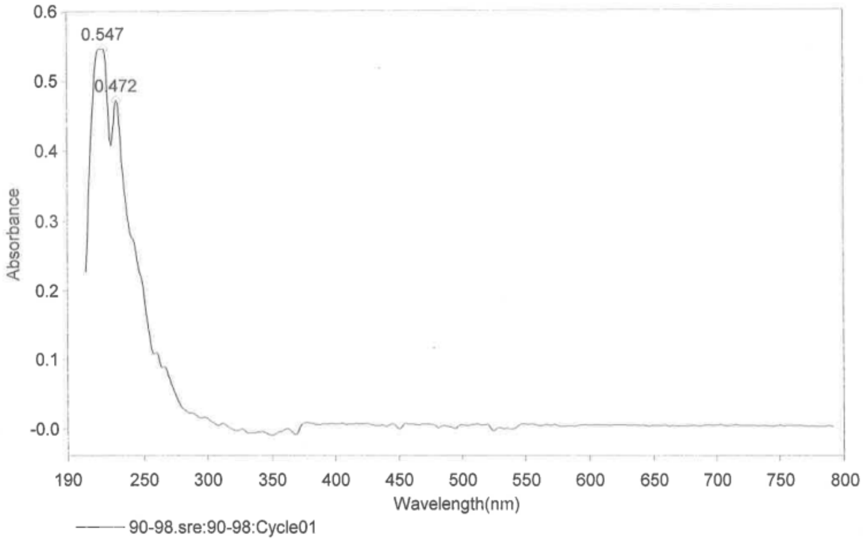

Results Table - 90-98.sre,90-98,Cycle01

nm	A	**Peak Pick Method**
219.00	0.547	Find 8 Peaks Above -3.0000 A
229.00	0.472	Start Wavelength190.00 nm
		Stop Wavelength800.00 nm
		Sort By Wavelength

Sensitivity Auto

Figure S10: UV/UV-Visible (MeOH) spectrum of compound **2**

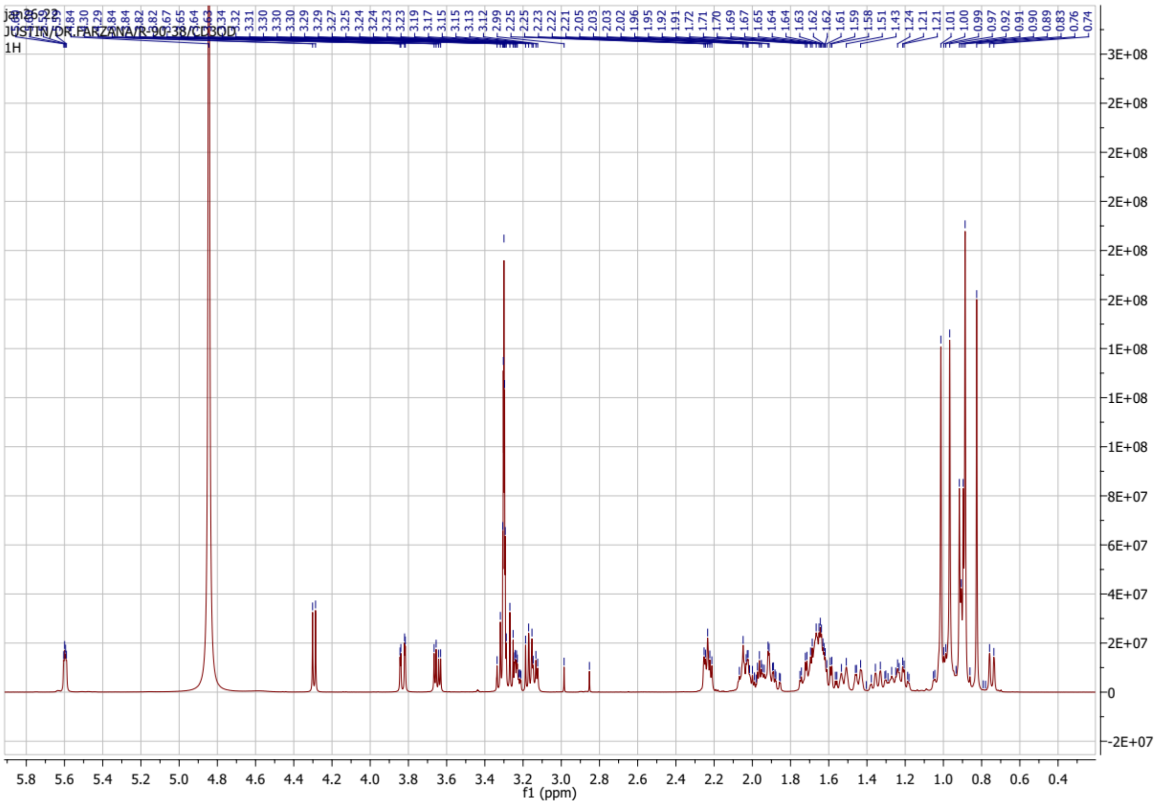

Figure S11: ^{1}H NMR (500 MHz, CD_3OD) spectrum of compound **2**

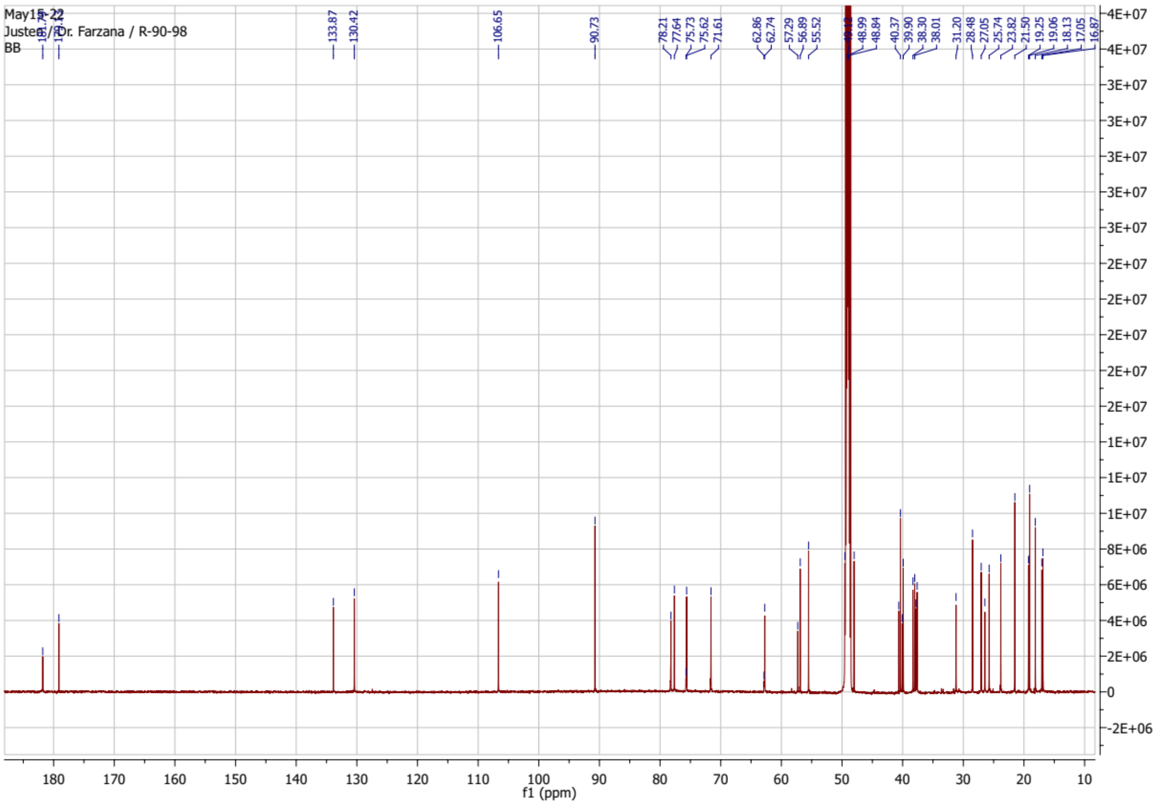

Figure S12: ^{13}C NMR (151 MHz, CD_3OD) spectrum of compound **2**

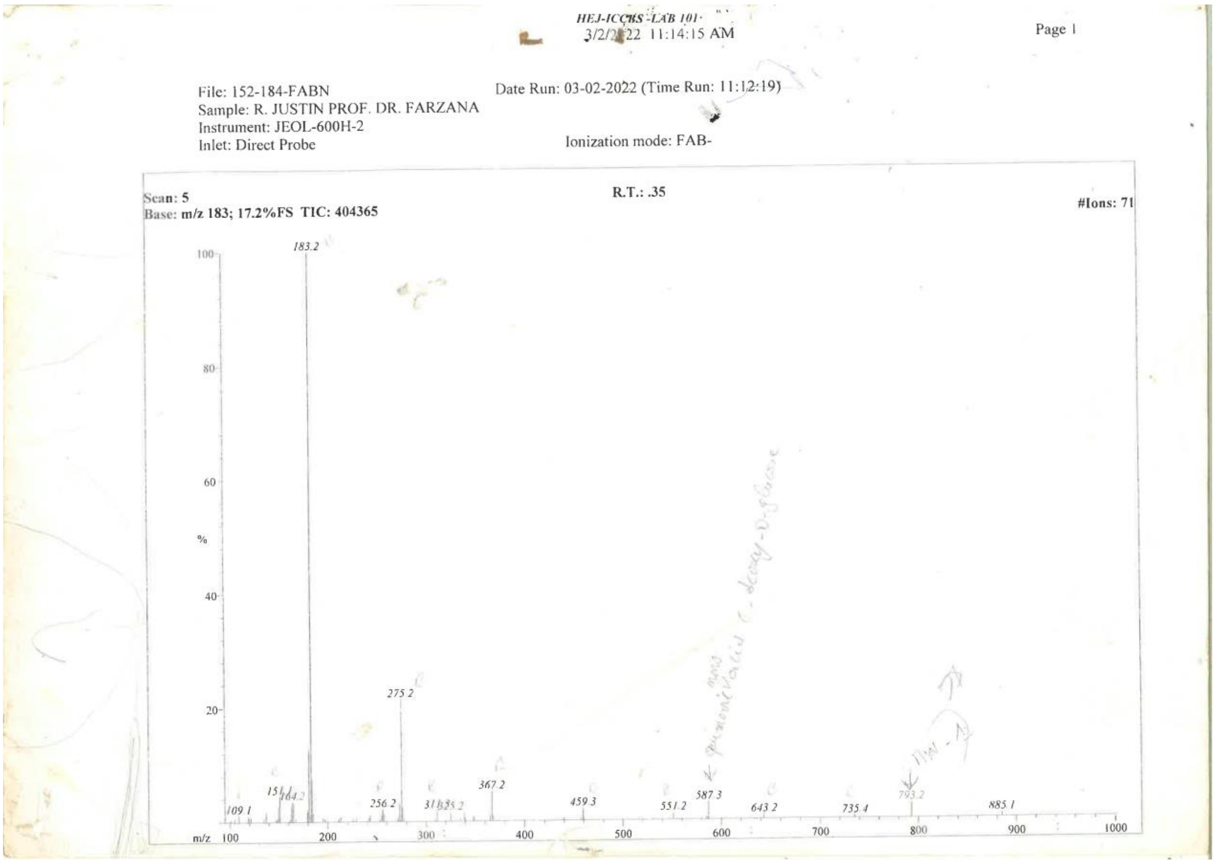

Figure S13: FABMS spectrum of compound **3**

HEJ-MASS LAB -ICCBS		JEOL HX 110 MASS SPECTROMETER (FAB-HR)		
STUDENT NAME	R. JUSTIN	SAMPLE CODE	DATE	7/3/2022
SUPERVISOR NAME	DR. FARZANA SHAHEEN	R-157-184	FAB (+VE / -VE)	+VE MODE

Measured Mass	Theoretical Mass	Delta [ppm]	Delta [mmu]	RDB	Composition
795.3114	795.311	0.4	0.4	35.5	C56 H43 O5
	795.3075	4.9	3.9	13.5	C38 H51 O18
	795.3169	-6.9	-5.5	26.5	C49 H47 O10
	795.3017	12.2	9.7	22.5	C45 H47 O13
	795.3228	-14.3	-11.4	17.5	C42 H51 O15
793.2958	793.2954	0.5	0.4	36.5	C56 H41 O5
	793.2919	4.9	3.9	14.5	C38 H49 O18
	793.3013	-6.9	-5.5	27.5	C49 H45 O10
	793.286	12.3	9.8	23.5	C45 H45 O13
	793.3071	-14.3	-11.3	18.5	C42 H49 O15

Figure S14: HRFABMS spectrum of compound **3**

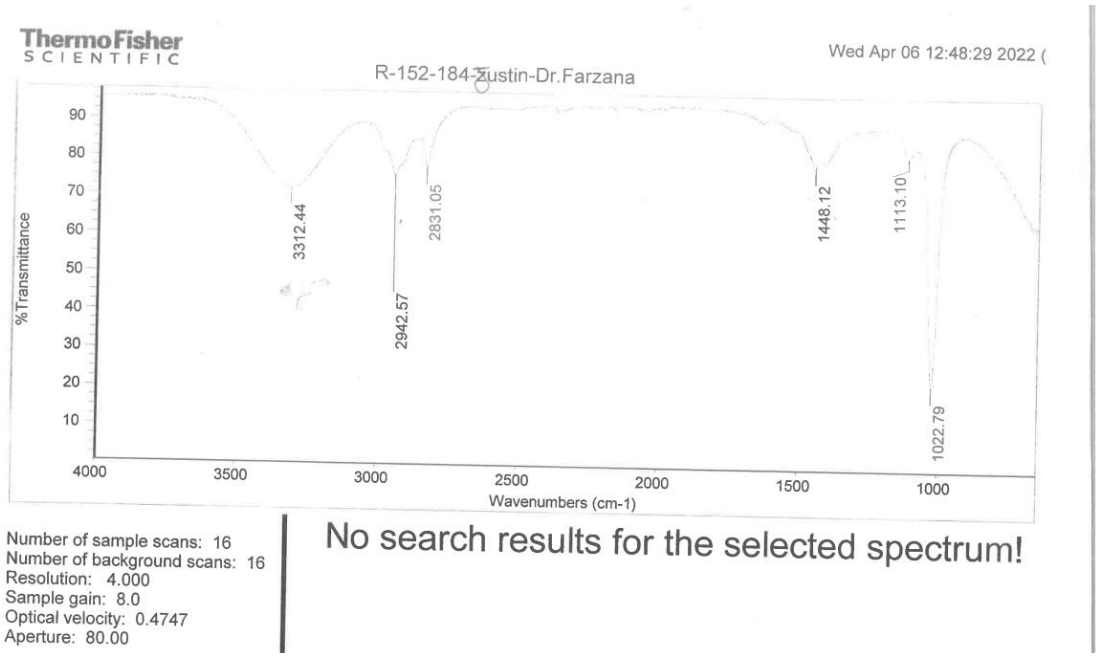

Figure S15: IR spectrum of compound **3**

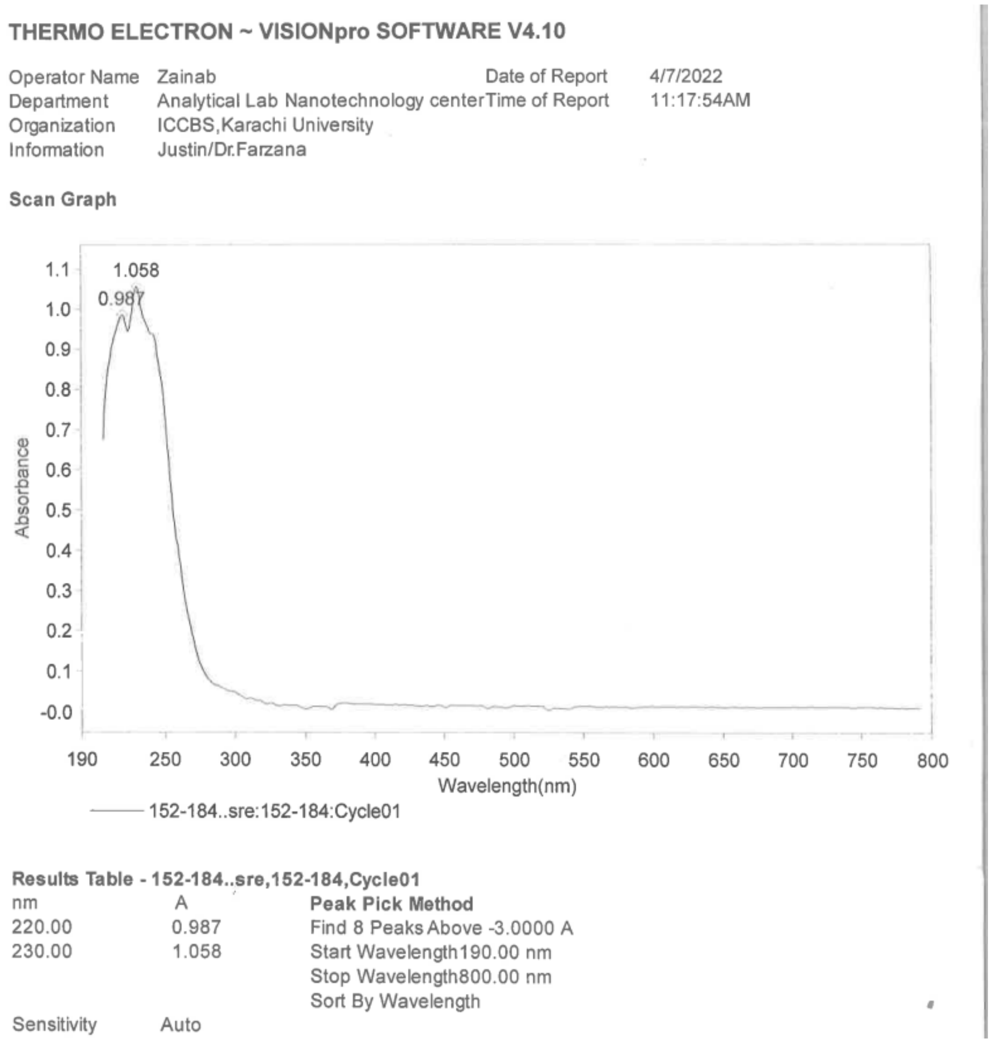

Figure S16: UV/UV-Visible (MeOH) spectrum of compound **3**

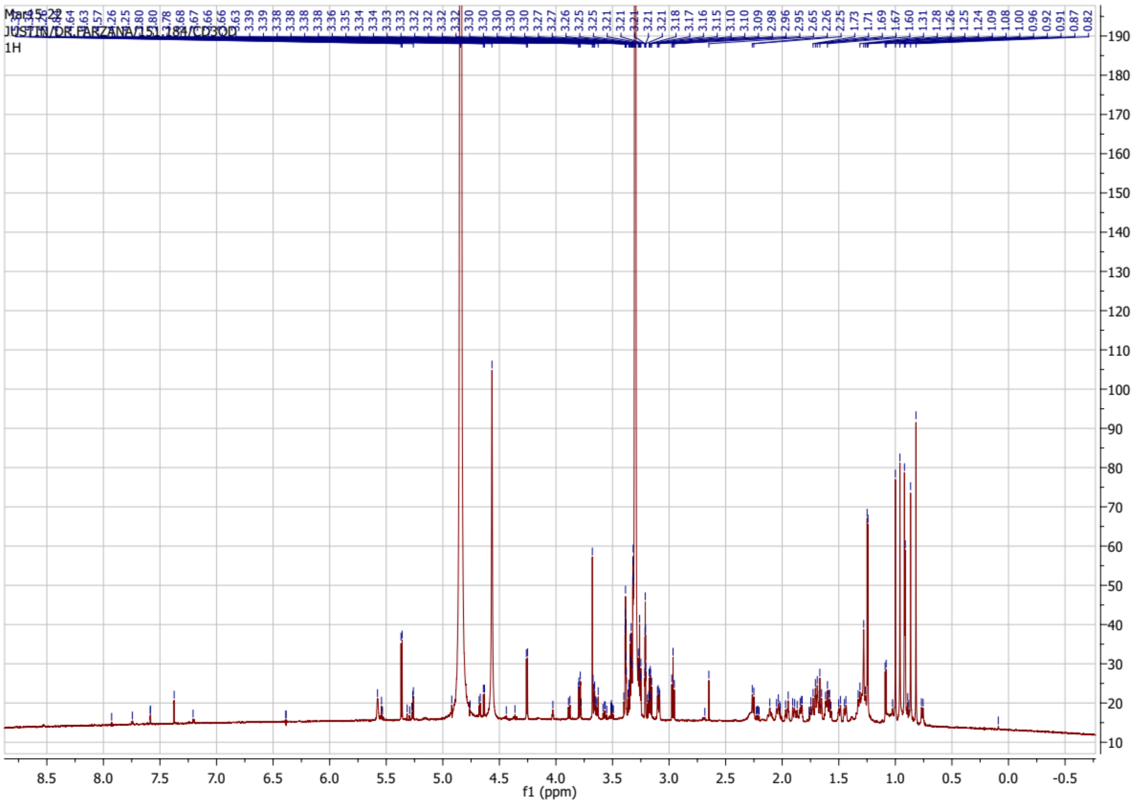

Figure S17: ^{1}H NMR (400 MHz, CD_3OD) spectrum of compound **3**

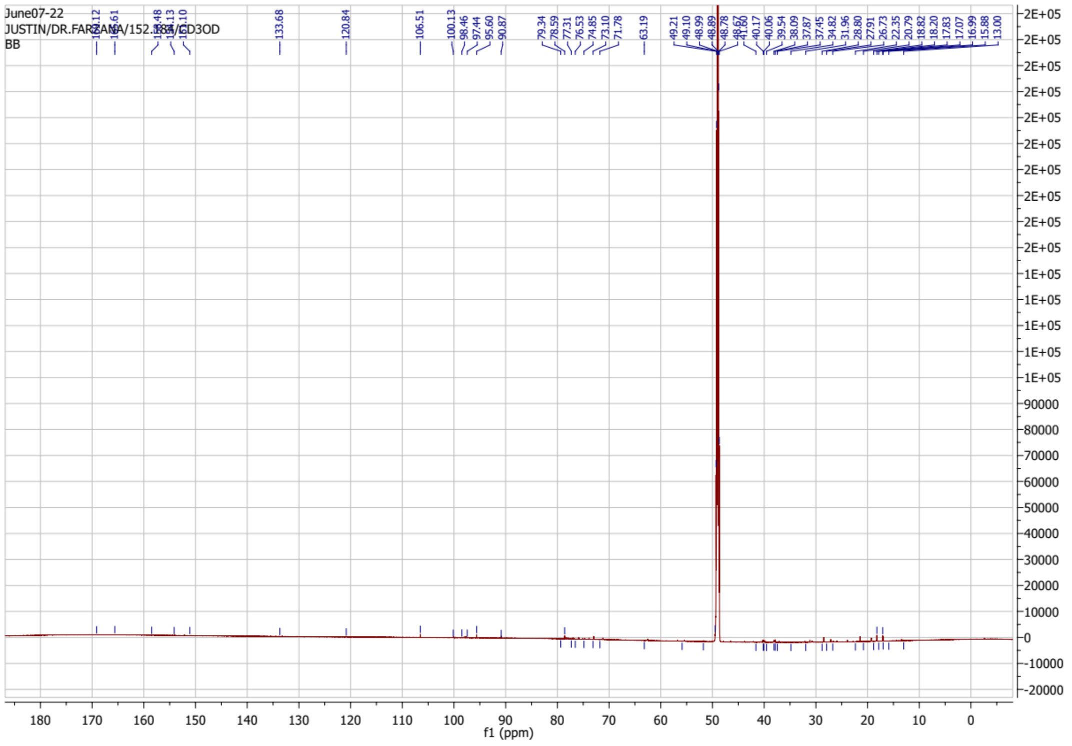

Figure S18: ^{13}C NMR (201 MHz, CD_3OD) spectrum of compound **3**

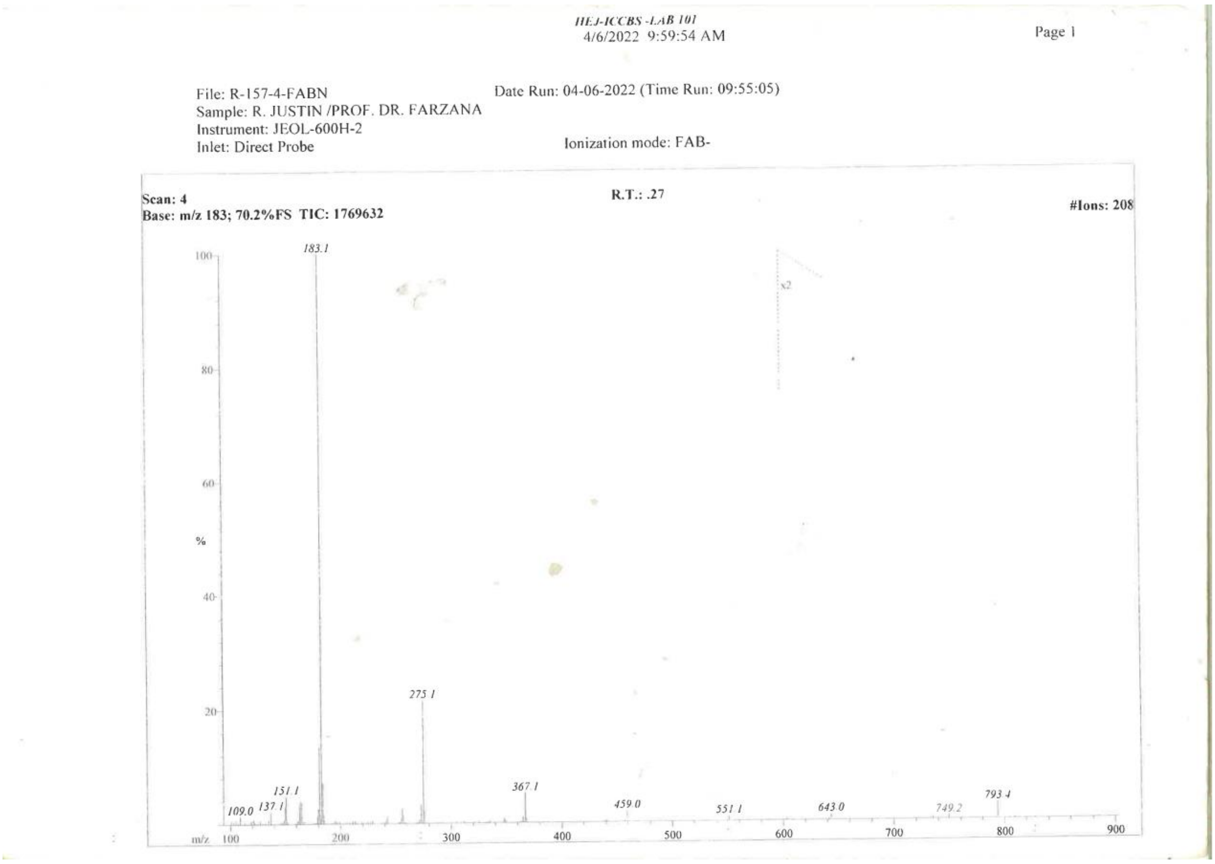

Figure S19: FABMS spectrum of compound **4**

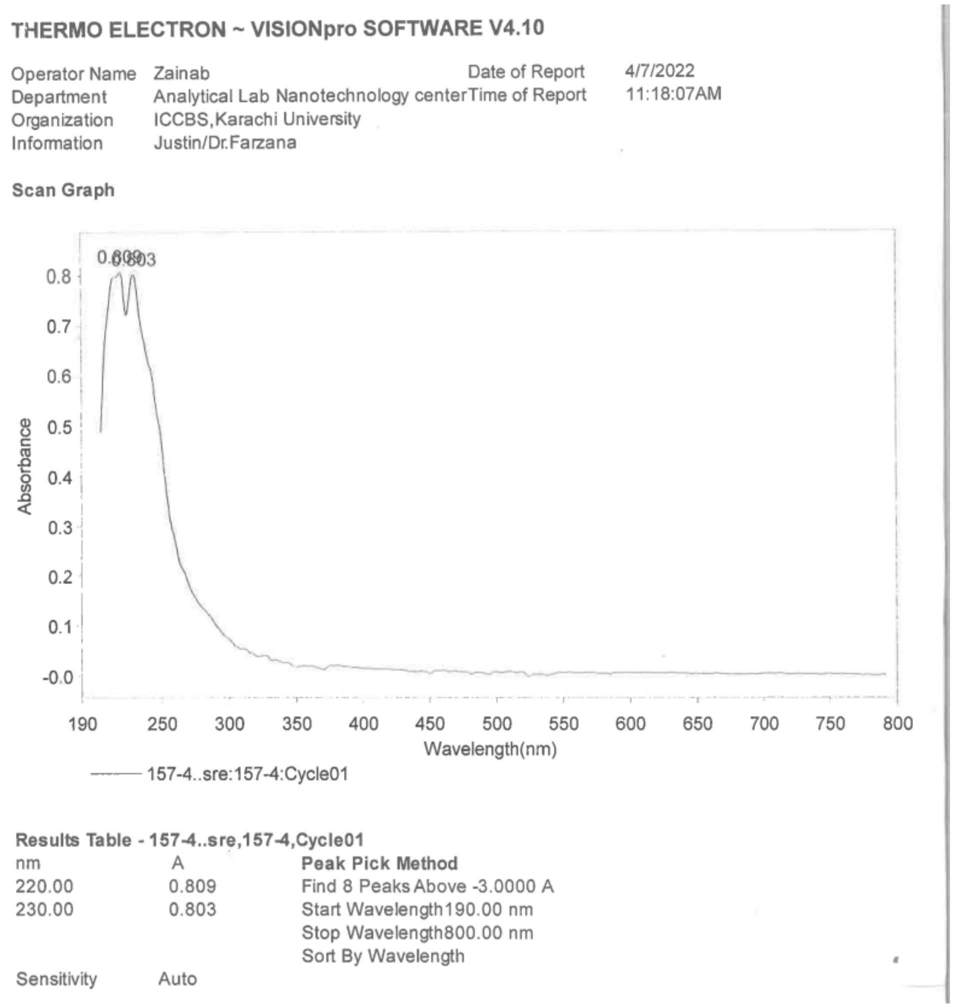

Figure S20: UV/UV-Visible (MeOH) spectrum of compound **4**

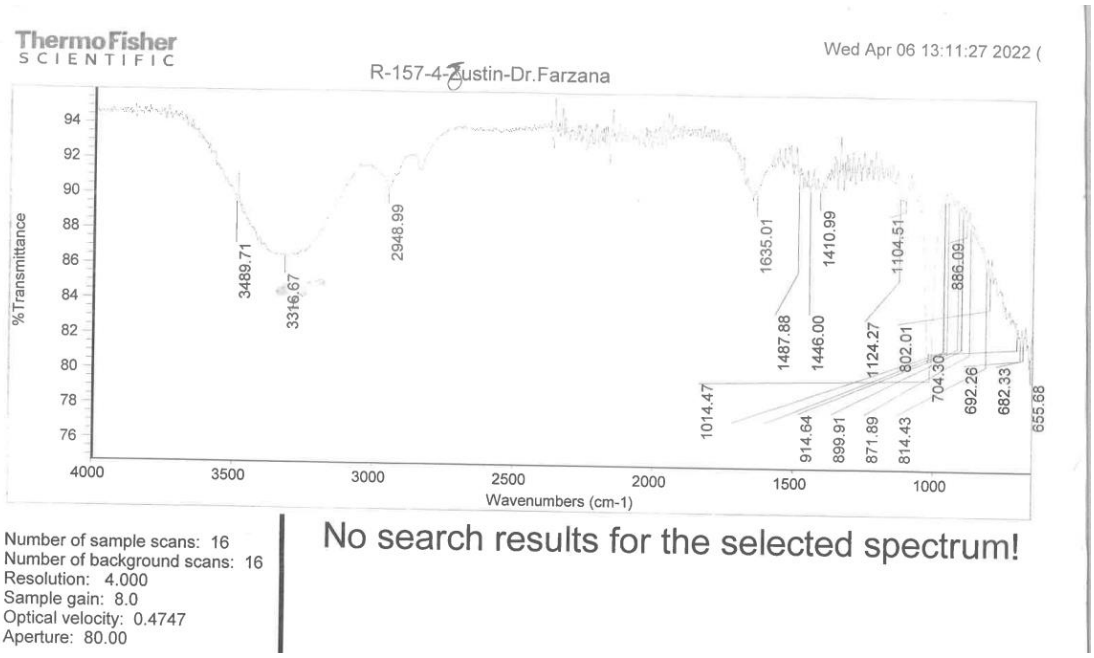

Figure S21: IR spectrum of compound **4**

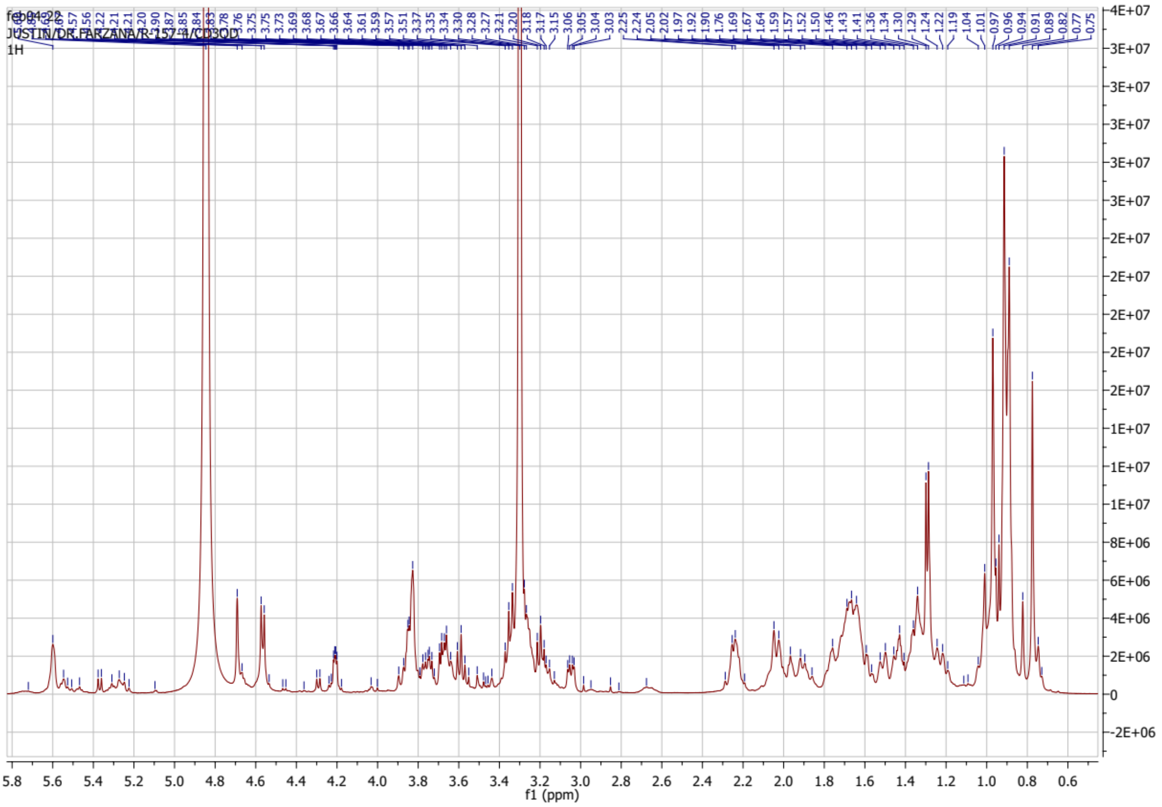

Figure S22: ^{1}H NMR (500 MHz, CD_3OD) spectrum of compound **4**

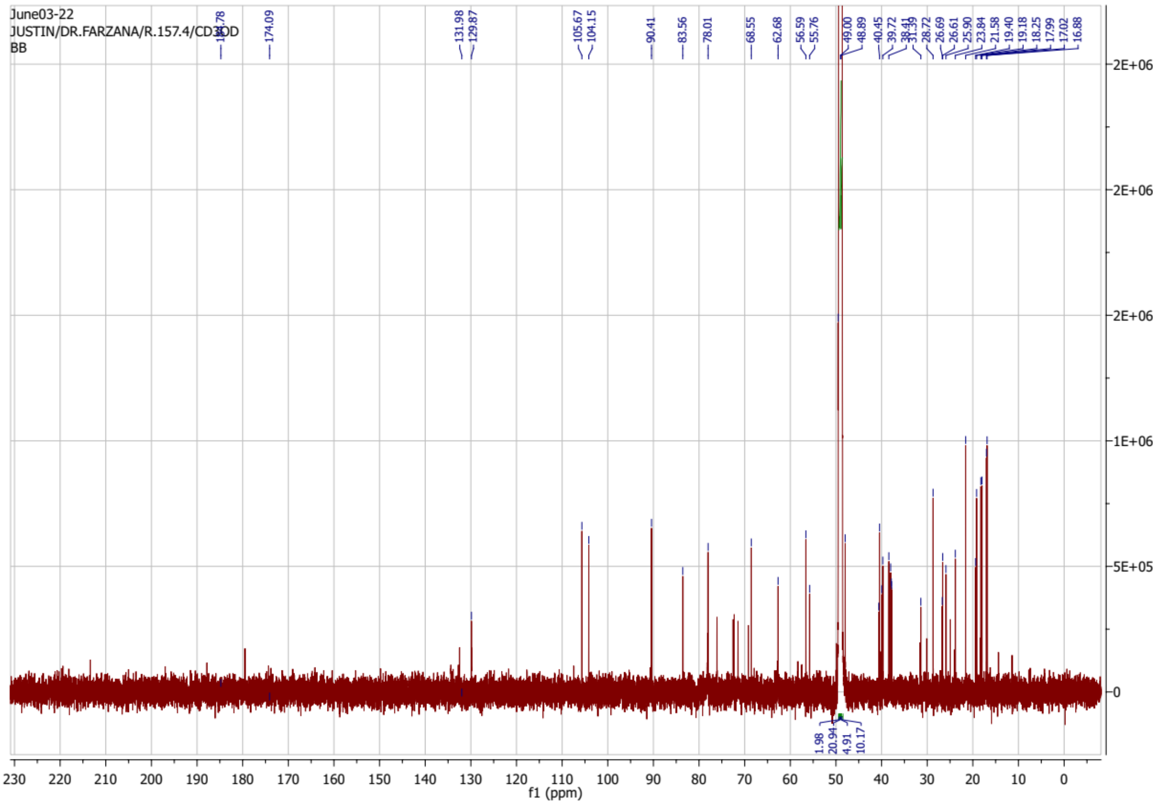

Figure S23: ^{13}C NMR (201 MHz, CD_3OD) spectrum of compound **4**

Table S1: *In-vitro* antiglycation activity fluorescence of *M. inermis* and *T. indica* plants fractions

Parts	Extracts Code	Concentrations	Fluorescence ± SD	% Inhibition ± SD
**Blank**	BSA-alone	NA	6.93 ± 0.09	NA
**AGEs Control**	Fructose-BSA	NA	81.30 ± 0.45	NA
**Positive Control**	Rutin	1 mM	7.53 ± 0.30	91 ± 0.80
M. inermis				
Leaf	Decoction	1mg/mL	13.93 ± 0.51	82.87 ± 0.63
		0.5mg/mL	17.93 ± 0.54	77.94 ± 0.66
		0.25mg/mL	22.88 ± 0.82	71.86 ± 1.01
	Ethyl acetate + Butanol + Acetone	1mg/mL	11.78 ± 0.14	85.50 ± 0.17
		0.5mg/mL	12.76 ± 0.16	84.30 ± 0.20
		0.25mg/mL	14.12 ± 0.08	82.63 ± 0.10
Stem	Ethyl Acetate + Acetone	1mg/mL	25.98 ± 0.63	68.04± 0.78
		0.5mg/mL	26.31 ± 0.88	67.64± 1.09
		0.25mg/mL	33.46 ± 4.74	61.95± 2.07
Root	Ethyl Acetate + Acetone	1mg/mL	25.73 ± 0.38	68.34± 0.47
		0.5mg/mL	28.67 ± 0.24	64.74± 0.29
		0.25mg/mL	37.41 ± 0.47	53.99± 0.58
T. indica				
Leaf	Ethyl Acetate + Butanol	1mg/mL	12.21 ± 0.21	84.97 ± 0.26
		0.5mg/mL	14.97 ± 0.61	81.59 ± 0.75
		0.25mg/mL	20.51 ± 0.65	74.77 ± 0.80
	Acetone	1mg/mL	8.41 ± 0.19	89.66 ± 0.23
		0.5mg/mL	9.98 ± 0.44	87.72 ± 0.55
		0.25mg/mL	15.29 ± 1.21	81.19 ± 1.49
Stem	Ethyl Acetate + Acetone	1mg/mL	6.65 ± 0.17	91.82± 0.21
		0.5mg/mL	7.64 ± 0.23	90.60± 0.28
		0.25mg/mL	9.65 ± 0.18	88.12± 0.23
Root	Ethyl Acetate + Acetone	1mg/mL	8.91 ± 0.21	89.03± 0.25
		0.5mg/mL	10.11 ± 0.01	87.56± 0.001
		0.25mg/mL	11.82 ± 0.37	85.45± 0.46
